# Organizational Factors Associated with Evidence-Based Practice Knowledge, Attitudes, and Implementation among Nurses in Saudi Arabia

**DOI:** 10.3390/ijerph19148407

**Published:** 2022-07-09

**Authors:** Naji Alqahtani, Kyeung M. Oh, Panagiota Kitsantas, Margaret Rodan, Adnan Innab, Saeed Asiri, Ali Kerari, Fayez Bin Hayyan, Mohammad Alharbi, Ghareeb Bahari

**Affiliations:** 1 Nursing Administration and Education Department, College of Nursing, King Saud University, P.O. Box 642, Riyadh 11421, Saudi Arabia; ainnab@ksu.edu.sa (A.I.); moalharbi@ksu.edu.sa (M.A.); gbahari@ksu.edu.sa (G.B.); 2 School of Nursing, College of Health and Human Services, George Mason University, Fairfax, VA 22030, USA; koh5@gmu.edu (K.M.O.); mrodan@gmu.edu (M.R.); 3 Department of Health Administration and Policy, College of Health and Human Services, George Mason University, Fairfax, VA 22030, USA; pkitsant@gmu.edu; 4 Medical Surgical Nursing Department, College of Nursing, King Saud University, P.O. Box 642, Riyadh 11421, Saudi Arabia; saasiri@ksu.edu.sa (S.A.); alikariri@ksu.edu.sa (A.K.); 5 Maternal and Child Health Nursing Department, College of Nursing, King Saud University, P.O. Box 642, Riyadh 11421, Saudi Arabia; fhayyan@ksu.edu.sa

**Keywords:** evidence-based practice, nurses, knowledge, attitudes, implementation, nursing leadership, work environment

## Abstract

Evidence-based practice (EBP) is crucial in keeping nurses aware of the current knowledge and improving clinical decision-making. The integration of nurses’ EBP competencies and organizational support has been suggested to create an effective arena in implementing EBP. The purpose of the study was to examine organizational factors influencing nurses’ EBP knowledge, attitudes, and implementation and identify staff nurses’ perceptions of EBP nursing leadership and hospital supports in Saudi Arabia. Data were collected from a convenience sample of staff nurses (N = 227) working in four hospitals using a cross-sectional, correlational descriptive design. Level of education (*p* < 0.05), EBP training (*p* < 0.05), unit type (ICU (*p* < 0.001) and ER (*p* < 0.01)), perceived nursing leadership (*p* < 0.001), and work environment (*p* < 0.05) supports were found significantly associated with nurses’ knowledge. Magnet recognition (*p* < 0.01) and knowledge (*p* < 0.001) had significant influence on nurses’ attitudes. Unit type (ER) (*p* < 0.05), knowledge (*p* < 0.001), and attitudes (*p* < 0.001) were associated with implementation. Encouragement to attend EBP trainings from nursing leadership was perceived by most nurses (51.1%). Nurses reported their hospitals support EBP through training (68.2%). Findings support the need for healthcare systems to create a culture that facilitates EBP implementation to enhance nurses’ EBP competencies and improve patients’ outcomes. Nursing managers may consider preparing nurses through education.

## 1. Introduction

Delivering quality care is the main goal of any healthcare organization. Evidence-based practice (EBP) has been recognized as a key driver for transforming healthcare and one of the fundamental provisions of quality healthcare and health outcomes improvement, including the quality of nursing practice [1]. EBP is crucial in keeping nurses aware of the current knowledge, improving clinical decision-making and judgment, and enhancing the engagement of patients in the decision-making process [2]. Additionally, nurses who base their practice on EBP use resources efficiently, increase patient satisfaction, and decrease inefficient or unneeded practices, which promotes more affordable and cost-effective care to clients’ and healthcare institutions [3].

Despite the benefits of EBP, there are challenges related to the implementation of EBP within the health care setting, which are related to nurses, healthcare institutions, and the EBP [4]. Research evidence indicated that EBP in nursing was influenced by organizational context, and the implementation of evidence to practice beyond the control of individual nurses as it relies on the nurses and organization leaders’ willingness to embrace and support EBP in the workplace [5].

It has been suggested that focusing on individual nurse decisions to implement EBP oversimplifies the issue [4]. However, this suggestion did not ignore the staff nurse’s contribution to EBP, it suggested that an encouraging workplace environment and effective nursing leadership empower staff nurses to participate in the EBP practice [4]. Magnet recognition is one of the organizational cultures that supports EBP, which facilitates the implementation of EBP [6]. However, Pryse found that Magnet recognition was not associated with increased implementation of EBP among U.S. nurses [4]. Yet, the integration of nurses’ EBP competencies and organizational support has been suggested to create an effective arena in implementing EBP [7,8].

In Saudi Arabia (SA), where this study took place, there has been increased attention to providing quality healthcare. In the past four decades, nursing services have been changing slowly since the majority of nurses were diploma holders compared to bachelor prepared nurses [9]. Bachelor prepared nurses receive more advanced research and EBP courses in comparison to diploma prepared nurses. Additionally, nursing education and practice are still in the infancy stage, and the need to improve nursing education and practice remains crucial [10]. However, in SA and other neighboring countries such as Jordan, Oman, and Iran, the implementation of EBP was found to be difficult for nurses and EBP has not been studied adequately [11,12,13,14,15]. Therefore, little is known about nurses’ EBP competencies.

**Figure 1:**
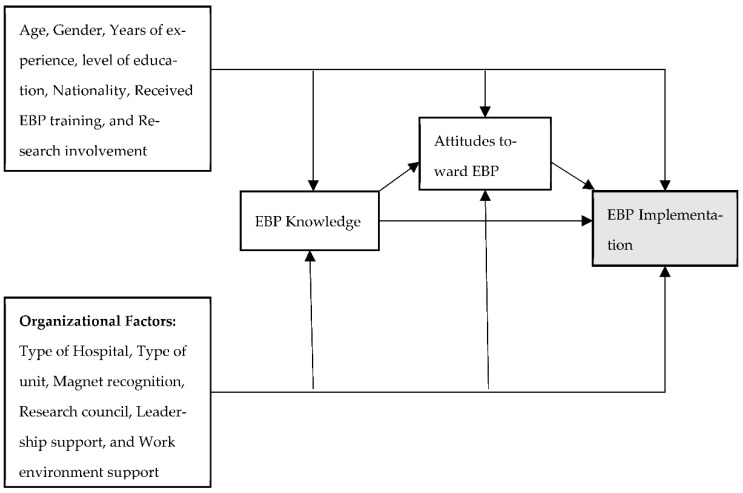
The study conceptual model.

The Saudi context is unique since there most nurses are expatriate nurses from different countries because of the shortage of Saudi nurses [16]. The majority of these nurses speak little to none of the native language of the county (Arabic) [17]. Additionally, they might not be familiar with the Saudi culture, which could have an impact on their practice which might ignore the cultural background of the patients’. Furthermore, the language and communication limitations, which are crucial for nursing care, could have an impact on nursing care [18]. In addition, the literature lacks studies that examine the organization factors associated with EBP in SA. Therefore, the aim of this study was: (1) To describe perceptions of nursing leadership and work environment support, knowledge, attitudes, and implementation of EBP among a sample of staff nurses in SA; (2) to identify factors influencing nurses’ knowledge, attitudes, and implementation regarding EBP based on the conceptual model of the study (Figure 1); and (3) to identify staff nurses’ perceptions of EBP nursing leadership and hospital supports from the two open-ended questions.

## 2. Materials and Methods

### 2.1. Theoretical Framework

The theory of Diffusion of Innovations developed by Rogers was used to guide this study [19]. Rogers defined diffusion as a process where innovation is communicated over time through members of a social system. Rogers defined innovation as a new idea, practice, or object of new occurrence. The theory explained that the innovation decision-making process occurs over five stages: knowledge, persuasion, decision, implementation, and confirmation.

In this study, EBP represented innovation. Organizations (hospitals) were the social system. Norms of the social system were related to the organizational support for EBP, which included nursing leadership and work environment. Moreover, knowledge was referred to nurses’ EBP knowledge. Persuasion was linked to nurses’ attitudes toward EBP. Finally, implementation was related to nurses’ EBP implementation. Figure 1 indicates the conceptual model being used in this study.

### 2.2. Study Design

The study utilized a cross-sectional, descriptive, correlational design.

### 2.3. Study Setting and Participants

From the accessible 5500 nurses in the study sites, G*Power software Version 3.1 (Heinrich-Heine-Universität, Düsseldorf, Germany) was utilized to calculate the needed sample size, which revealed a minimum of 171 participants to run the statistical analysis. A convenience sample from all registered nurses who provided care in inpatient units and had at least six months of experience in one of the selected hospitals were eligible to participate. A total of 302 questionnaires were distributed, with 227 returned, representing a response rate of 75.2%. The study was conducted in four different hospitals in Riyadh, SA. The hospitals included in the study were (1) public non-Ministry of Health (MOH), Magnet recognized hospital, (2) public MOH, non-Magnet hospital, (3) teaching hospital, non-Magnet, and (4) private, non-Magnet, hospital.

### 2.4. Data Collection Procedures

Institutional Review Boards (IRBs) approvals were obtained by the researchers. After IRBs approvals, communications with hospital directors and Chief Nursing Officers in the selected hospitals were initiated to grant permission to discuss the study at the next nurse manager meeting with staff nurses. Without the presence of the nurse managers, the researcher met the staff nurses between July and August 2018. The researcher described the study to the participants. Next, informed consent and paper-pencil questionnaires were administered. Those who consented to participate were requested to fill out the questionnaire on-site and return them to the researcher.

#### Measures

A self-reported, five-part paper-pencil questionnaire was utilized. The first part gathered the sample characteristics information (age, gender, nationality, years of experience, level of education, received EBP training, and research involvement) and four items related to the organizational factors (type of hospital, Magnet recognition, type of unit, and research and EBP council familiarity). The second part included the Evidence-based Practice Questionnaire (EBPQ) [20]. The third and fourth parts included the EBP nursing leadership and EBP work environment scales [21]. The last part included two open-ended questions. The two open-ended questions were: (1) “How does your nurse manager at the unit level support EBP in your unit?” (2) “How does your hospital provide support for EBP?” Permissions to use all of the scales were granted from the authors.

Staff nurses’ EBP implementation, attitudes toward EBP, and EBP knowledge (referred hereafter as implementation, attitudes, and knowledge, respectively) were measured using the EBPQ [20]. It has 24 items with three subscales: frequency of practice (6 items), attitudes toward EBP (4 items), and knowledge (14 items). It has good internal consistency with overall Cronbach’s α of 0.87, the Cronbach’s α for each subscale ranged from 0.79 to 0.91. All items’ scores had a range of 1–7, a higher score indicating more knowledge, higher attitudes, and more implementation of EBP. In this study, the internal consistencies were excellent (Cronbach’s α = 0.95 for the scale). For each subscale, the Cronbach’s α ranged from 0.83–0.96.

Staff nurses’ perceptions of nursing leadership support of EBP (referred hereafter as nursing leadership support) were measured using the EBP nursing leadership scale [21]. It is a unidimensional scale that has 10 items and can be measured using a 5-point Likert scale with a range of 1 (strongly disagree) to 5 (strongly agree). Higher scores indicate more EBP nursing leadership support. It has excellent internal consistency with Cronbach’s α of 0.96 [21]. In this study, the reliability was similar (Cronbach’s α = 0.96).

Staff nurses’ perceptions of work environment support for EBP (referred hereafter as work environment support) were measured using the EBP work environment scale [21]. It is a unidimensional scale that has eight items and can be measured using a 5-point Likert scale with a ranging of 1 (strongly disagree) to 5 (strongly agree). Higher scores indicate more EBP organizational support. It has good internal consistency with Cronbach’s α of 0.86 [21]. In this study, the internal consistency was higher (Cronbach’s α = 0.90).

### 2.5. Ethical Considerations

Prior to data collection, IRBs permissions were granted. The researcher emphasized the voluntariness and ensured to maintain the participant’s privacy, confidentiality, and anonymity. Informed consent was obtained by the participant’s willingness to complete and return the questionnaires.

### 2.6. Data Analysis

Data analyses were performed using the Statistical Package for Social Sciences (SPSS) version 24.0 software (Armonk, NY, USA) [22]. Descriptive statistics were conducted to evaluate frequencies and percentages for the categorical variables, and central tendency for the continuous variables. Pearson’s *r* correlations were performed to examine the relationships between continuous variables. To assess any significant differences in the average scores of the EBP measures, *t*-test, and one-way analysis of variance (ANOVA) were performed for each of the categorical independent variables.

The outcome variables in the study were knowledge, attitudes, and implementation. Therefore, hierarchical multiple linear regression models were built to examine the effects and variance of the organizational factors explained in knowledge, attitudes, and implementation after controlling for the sample characteristics. The predictor variables were entered based on the conceptual model of this study (Figure 1). In the first step to predict the outcome variables, sample characteristics were entered. Organizational factors were entered in the second step. For the model predicting attitudes, knowledge was entered at the last step. Further, to predict implementation, the first two steps of the regression predicting knowledge and attitudes were repeated. Next, knowledge and attitudes were inserted into the regression model in the final step.

Content analysis was conducted by counting, comparing, and grouping common words into meaningful categories from the narrative responses to the open-ended questions [23].

## 3. Results

### 3.1. Sample Characteristics

Table 1 displays the sample characteristics and organizational factors. The mean age was 33.5-year-old (SD = 6.7). Most of the participants (85.5%) were females. Only 7% of the nurses were Saudis. The years of experience was an average of 10.5 years (SD = 6.4). More than 71% of the participants held a bachelor of science in nursing (BSN) or higher degrees. Over 32% of the participants worked in a teaching hospital. Only 20.3% of the participants were working in a Magnet recognized hospital. Nearly half of the participants (45.8%) were providing care in general units. Over 67% of the nurses had attended EBP training during their careers. Additionally, only 37.4% of the nurses were involved in research activities. Most staff nurses (77.5%) knew that their hospital had a Research and EBP Council. The average nursing leadership support perception score was 38.2 (SD = 7.00). Further, the average perception of the EBP work environment support score was 29.2 (SD = 5.4). EBP attitudes showed the highest average scores, followed by EBP knowledge scores, and EBP implementation scores (Table 2).

### 3.2. The Associations of Nursing Leadership and Work Environment Supports, Knowledge, Attitudes, and Implementation

Table 2 shows the correlations of the main variables of the study. There were positive correlations between nursing leadership and work environment supports (*r* = 0.671, *p* < 0.001), knowledge (*r* = 0.405, *p* < 0.001), attitudes (*r* = 0.325, *p* < 0.001), and implementation (*r* = 0.300, *p* < 0.001). Work environment support was positively associated with knowledge (*r* = 0.384, *p* < 0.001), attitudes (*r* = 0.257, *p* < 0.001), and implementation (*r* = 0.302, *p* < 0.001). Additionally, the associations between implementation and knowledge were moderately positive (*r* = 0.545, *p* < 0.001). Similar associations were found between implementation and attitudes (*r* = 0.460, *p* < 0.001).

### 3.3. Differences in Nursing Leadership and Work Environment Supports, Knowledge, Attitude, and Implementation by Sample Characteristics and Organizational Factors

Table 3 shows that female nurses had better perceptions of EBP work environment support than male nurses (*p* < 0.01). However, gender did not show any significant difference in nursing leadership support, knowledge, attitudes, and implementation. There were significant differences in nursing leadership support (*p* < 0.01), work environment support (*p* < 0.05), and attitudes (*p* < 0.001) among the four hospitals. Using the Bonferroni post-hoc test, nurses who worked in a non-MOH, Magnet hospital had better perceptions of nursing leadership support compared to nurses working in a MOH public hospital (*p* < 0.05) and teaching hospital (*p* < 0.01). However, there was no significant differences between the non-MOH, Magnet hospital and the private hospital. Regarding work environment support, a significant difference was found between staff nurses working in a non-MOH, Magnet hospital and MOH, public hospital only (*p* < 0.05). Non-MOH, Magnet hospital nurses had higher perceptions of work environment support than MOH hospital nurses. Non-MOH, Magnet hospital nurses had better attitudes compared to MOH, public hospital nurses (*p* < 0.001) and private hospital nurses (*p* < 0.05). However, no significant difference was found between non-MOH, public and teaching hospital nurses.

Nurses working in a Magnet recognized hospital were significantly different than nurses working in non-magnet recognized hospitals in perceived nursing leadership support (*p* < 0.01), work environment support (*p* < 0.05), and attitudes (*p* < 0.01). Magnet hospital nurses had higher perceptions of nursing leadership and work environment supports and more positive attitudes than nurses working in non-Magnet hospitals. Additionally, there were significant differences among unit types in knowledge (*p* < 0.001) and implementation (*p* < 0.01). Using the Bonferroni post-hoc test, nurses working in the ICU (*p* < 0.001) and ER (*p* < 0.05) had better knowledge than general units’ nurses. However, there was not any significant difference between ICU and ER nurses in their knowledge. Regarding implementation, ER nurses implemented EBP more than nurses in general units significantly (*p* < 0.01). Nevertheless, no significant difference was found in implementation between ER and ICU nurses.

A significant difference was found in knowledge between nurses who received EBP training and nurses who had never received training (*p* < 0.01). Nurses who received training had better knowledge. Similarly, a significant difference was discovered in knowledge between nurses involved in research activities and their counterparts (*p* < 0.01). Nurses who were familiar with the Research and EBP council in their hospitals had better perceptions of nursing leadership support (*p* < 0.001), work environment support (*p* < 0.01), knowledge (*p* < 0.01), and attitudes (*p* < 0.01). However, no significant difference was found in implementation.

### 3.4. Multiple Linear Regression Analyses

Hierarchical multiple linear regression analyses were conducted using sample characteristics and organizational factors, knowledge, attitudes, and implementation (Table 4). In the first step of the model predicting knowledge, EBP training and research involvement (both *p* < 0.05) were significant and accounted for 7.0% of the total variance (*p* < 0.05). In the final step, level of education–diploma (*p* < 0.05) was negatively associated with knowledge. Nurses with a diploma had significantly lower levels of knowledge compared to those with a BSN degree or higher. In the last step, EBP training (*p* < 0.05), ICU unit (*p* < 0.001), ER unit (*p* < 0.01), nursing leadership support (*p* < 0.001), and work environment support (*p* < 0.05) had positive associations with knowledge. The organizational factors contributed 27.7% to the total variance (*p* < 0.001). The total explained variance in the knowledge model by all variables was 34.7% (*p* < 0.001).

In the model predicting attitudes, only Magnet recognition (*p* < 0.05), ICU unit (*p* < 0.05), and nursing leadership support (*p* < 0.01) had a positive relationship from the organizational factors at the second step. The organizational factors contributed 15.0% to the total explained variance (*p* < 0.001) in attitudes. In the last step, only Magnet recognition (*p* < 0.01) remained significant from the organizational factors after adding knowledge to the model. Knowledge was correlated with attitudes positively (*p* < 0.001) and added 4.9% of the variance in attitudes. The total explained variance in attitudes toward the EBP model was 21.7% (*p*< 0.001).

In the model predicting EBP implementation, ICU unit (*p* < 0.05), ER unit (*p* < 0.01), nursing leadership support (*p* < 0.05) were significantly associated with the outcome in the second step. Organizational factors contributed 15.8% to the total variance. In the final step, only ER unit (*p* < 0.05) remained significant from the organizational factors after adding knowledge and attitudes to the model. Knowledge (*p* < 0.001) and attitudes (*p* < 0.001) had positive associations with the implementation. Knowledge and attitudes contributed 24.6% to the variance (*p* < 0.001). The overall model explained 42.9% of the variance in implementation.

### 3.5. EBP Nursing Leadership and Hospital Supports

Thirty-eight percent of participants (*n* = 88) responded to the open-ended questions. More than 51% of the 88 participants stated that their nurse manages EBP support in the unit by encouraging them to attend EBP training, while 35.2% of the respondents indicated that their managers support EBP by conducting practice meeting updates, offering rewards (5.7%), monitoring practice (2.3%), and creating a journal club (1.1%). However, 4.5% of the participants stated that their nurse managers did not support EBP in their units.

Regarding hospital support for EBP, sixty participants (68.2%) reported their hospitals support EBP through training, providing the necessary resources (11.4%), monitoring practice change (5.7%), encouraging attending EBP training and changing practice (3.4%), providing financial support for EBP training and conferences (3.4%), and dissemination of EBP in the hospital (1.1%). Nevertheless, 6.8% of the participants stated that their hospitals did not support EBP.

## 4. Discussion

The findings revealed that nurses in SA had moderate perceptions of nursing leadership and work environment supports. Similar findings were reported among nurses in the USA [4]. Nurses in SA had positive attitudes toward EBP and acceptable knowledge. However, their EBP implementation had the lowest mean. This was in line with previous studies that examined nurse EBP knowledge, attitudes, and usage in different countries [12,14,24,25]. Yet, the findings were inconsistent with a Saudi study that had more diploma prepared nurses [11], which might contributed to the difference in EBP competencies.

The findings showed that a bachelor level of education or higher influenced staff nurses’ knowledge. Nurses with BSN degrees or higher had better knowledge than diploma prepared nurses. Bachelor’s degree programs encompass more courses about research and EBP than diploma degree programs, which might contribute to the difference. This finding was consistent with two studies that were conducted in SA and other previous studies that were conducted in Jordan, Iran, and USA, which indicated a higher level of education influenced nurses’ knowledge [12,15,24,26]. However, the level of education did not influence nurses’ attitudes and implementation. These findings were contrary to previous studies that indicated that higher levels of education were related to more positive attitudes and better implementation among nurses in SA, Jordan, Iran, and USA, respectively [6,11,12,15,24,26,27,28]. The finding that the level of education was not associated with nurses’ attitudes and implementation might be due to the barriers all nurses face in the hospitals despite their education level. Another reason might be related to the education they received, which might not focus on the EBP value.

Receiving EBP training improved nurses’ knowledge about EBP. This finding was inconsistent with previous studies that examined the association between attending EBP training and nurses’ knowledge in SA, USA, Italy, and Singapore, respectively [11,24,29,30,31]. However, EBP training was not associated with nurses’ attitudes and implementation. Previous studies in SA and USA found similar results [11,24]. Our findings suggest that EBP training improved knowledge because it involved practicing the steps of EBP development. Nonetheless, EBP training did not increase nurses’ attitudes and implementation of EBP in the practice. Consequently, healthcare institutions might need to review training to include aspects that might enhance attitudes and practice of EBP and also focus on qualities that influence nurses’ engagement of the process in their activities.

Working in a Magnet recognized facility was not related to nurses’ knowledge and implementation. Similarly, Pryse found that Magnet recognition did not impact nurses’ EBP implementation in the USA [4]. This finding suggested that the implementation was challenging even in Magnet environments. However, nurses who provided care in Magnet hospitals had more positive attitudes than nurses who provided care in non-Magnet hospitals. This could be because Magnet facilities provide the necessary infrastructure and more organizational support for EBP [32]. In addition, nurses working in a Magnet hospital had better perceptions of nursing leadership and work environment support, which may have influenced their attitudes. Previous studies indicated that nurses working in Magnet institutions perceived fewer barriers to EBP use [6,24].

The current study indicated that nurses providing care in ICU or ER had higher knowledge than nurses in general units. In addition, nurses working in ER had higher implementation scores than other units. This might be related to the complex nature of critical care units which requires nurses to keep their knowledge updated to the latest evidence by attending advanced courses such as Advanced Cardiac Life Support (ACLS). Their attendance in advanced courses may have increased their awareness about the importance of the latest evidence. Previous studies indicated that nurses providing care in critical care units in Jordan and Iran had higher knowledge and attitudes [12,15]. However, in this study, unit type did not influence nurses’ attitudes toward EBP. The reason might be due to the work environment and the amount of support all nurses perceived. In this study, perceived EBP nursing leadership and work environment supports were not statistically different based on the type of unit.

Perceptions of nursing leadership and work environment supports were correlated with knowledge, attitudes, and implementation in the bivariate analysis. However, they were correlated with knowledge only in the multivariate analysis. The reason might be related to the fact the nurses were encouraged to attended training and their hospitals provided the necessary training, which might have improved their knowledge. Therefore, we found that EBP training influenced nurses’ knowledge only in this study. Furthermore, nursing leadership support was not associated with attitudes after controlling knowledge. Similarly, nursing leadership and work environment supports were not associated with implementation after controlling knowledge and attitudes. In a US study, Pryse found that nursing leadership and work environment supports were not associated with implementation [4]. On the contrary, Melnyk and colleagues found that organizational culture for EBP was related to implementation [33]. Our findings suggest that implementation was problematic, and organizations might need to consider nurses as individuals to increase their engagement in the process.

Knowledge and attitudes regarding EBP influenced implementation positively more than the perceived supports from nursing leadership and work environment. Similarly, previous findings from the U.S. and SA indicated knowledge and attitudes influenced implementation [34,35]. However, these studies did not examine staff nurses’ perceptions of organizational support for EBP. Our findings suggest that organizations might need to improve nurses’ knowledge and attitudes regarding EBP to increase nurses’ engagement in the EBP process.

Most of the nurses in this study reported that their nurse managers support EBP through encouragement to attend EBP training. Leadership support for EBP, such as managers coaching and positive reinforcement, has been linked to the facilitation and enhancement of the use of EBP among U.S. nurses [36,37]. Additionally, the majority of nurses indicated that their hospitals support EBP by providing EBP trainings. Hospital work environment support for EBP in the form of education, hands-on training, and a culture that encourages EBP were determinants of EBP supportive environment [38]. Our study was one of the few studies that examined staff nurses’ perceptions of EBP nursing leadership support and work environment support and their relations with nurses’ EBP competencies. Therefore, further research is recommended.

### Limitations

The design of the study, cross-sectional, has its limitations due to the collection of the data at one point in-time. Therefore, a true cause and effect relationship may not be possible. The generalizability of the findings might be affected since the study utilized the convenience sampling method, which might affect the outcome. Furthermore, the data collection from four large hospitals in one urban city, where nurses have a higher level of education, may not represent the perceptions of staff nurses in other cities, particularly rural cities. Moreover, the study did not assess the nationalities of the expatriate nurses. Therefore, future research in this regard should concern a more diverse group of nurses from different areas (urban and rural) and with different levels of education. The use of self-reported questionnaires has its limits, such as socially desirable and/or false responses. Individual staff nurses’ perceptions about the organization may be limited and they may not know all the available resources. Therefore, their reflections of the support may not reflect all aspects of the organizational work environment.

## 5. Conclusions

Our findings supported the need for healthcare systems to create cultures that facilitate EBP by considering nurses as individuals to enhance their attitudes towards the importance of EBP and the capability to implement it to improve patients’ outcomes as well as to enhance nurses’ EBP competencies. Nursing directors, educators, and hospital directors in SA should use the study findings to assist nurses overcome EBP barriers and benefit from EBP by improving their knowledge, attitudes, and implementation of EBP.

The study provided evidence that nurses’ EBP knowledge and attitudes would encourage them to implement EBP more than the support they perceived from their nursing leadership and work environment. Therefore, investing in improving their knowledge and attitudes toward EBP through continuous education and providing the resources and support they need would likely improve the implementation of EBP, which is the desired outcome for the healthcare institutions.

## Figures and Tables

**Table 1:** Sample characteristics and organizational factors (N = 227).

Variable	n (%) or Mean ± SD (Range)
Age	33.55 ± 6.70 (23–59)
Gender	
Male	33 (14.5)
Female	194 (85.5)
Nationality	
Saudi	16 (7.0)
Non-Saudi	211 (93.0)
Years of experience	10.54 ± 6.37 (1–36)
Level of Education	
Diploma	64 (28.2)
BSN or higher	163 (71.8)
Hospital Type	
Non-MOH, Public	46 (20.3)
MOH, Public	54 (23.8)
Teaching	73 (32.2)
Private	54 (23.8)
Magnet recognition	
Yes	46 (20.3)
No	181 (79.7)
Unit Type	
General units	104 (45.8)
ICU	52 (22.9)
ER	71 (31.3)
Received EBP training	
Yes	154 (67.8)
No	73 (32.2)
Research involvement	
Yes	85 (37.4)
No	142 (62.6)
Research and EBP Council familiarity	
Yes	176 (77.5)
No	51 (22.5)

**Table 2:** Correlation matrix and descriptive statistics for perceived nursing leadership and work environment support for EBP, knowledge, attitudes, and implementation (N = 227).

Variables (Possible Score Range)		1	2	3	4	5	Range	Mean (SD)
1	Nursing leadership support (10–50)	1					11–50	38.27 (7.00)
2	Work environment support (8–40)	0.671 **	1				16–40	29.22 (5.45)
3	Knowledge (1–7)	0.405 **	0.384 **	1			1.36–6.93	4.72 (0.96)
4	Attitudes (1–7)	0.325 **	0.257 **	0.357 **	1		1–7	4.82 (1.49)
5	Implementation (1–7)	0.300 **	0.302 **	0.545 **	0.460 **	1	1–7	4.36 (1.53)

** *p* < 0.001.

**Table 3:** Differences in perceived nursing leadership and work environment support, knowledge, attitude, and implementation regarding EBP by sample characteristics and organizational factors (N = 227).

Variables		Nursing Leadership Support		Work Environment Support		Knowledge		Attitudes		Implementation	
Total Possible Range		10–50		8–40		1–7		1–7		1–7	
	Range	Mean (SD)		Mean (SD)		Mean (SD)		Mean (SD)		Mean (SD)	
**Age**	23–59		*r* = 0.065		*r* = 0.094		*r* = 0.081		*r* = 0.064		*r* = 0.039
**Gender**	Male Female	37.33 (6.85) 38.43 (7.03)	*t* = 0.830	26.91 (5.80) 29.61 (5.30)	***t*** **= 2.665 ^b^**	4.67 (0.95) 4.72 (0.99)	*t* = 0.287	4.62 (1.34) 4.85 (1.52)	*t* = 0.828	4.22 (1.44) 4.38 (1.55)	*t* = 0.534
**Nationality**	Saudi Non-Saudi	39.75 (7.06) 38.16 (7.00)	*t* = 0.877	28.88 (7.62) 29.24 (5.27)	*t* = −0.259	4.80 (1.01) 4.71 (0.99)	*t* = 0.357	5.03 (1.46) 4.80 (1.50)	*t* = 0.584	4.42 (1.70) 4.35 (1.52)	*t* = 0.156
**Years of experience**	1–36		*r* = 0.002		*r* = 0.038		*r* = 0.050		*r* = 0.039		*r* = 0.058
**Level of Education**	Diploma BSN or higher	38.58 (5.94) 38.15 (7.38)	*t* = 0.417	30.13 (4.67) 28.86 (5.70)	*t* = 1.580	4.71 (1.02) 4.72 (0.98)	*t* = −0.085	4.82 (1.29) 4.82 (1.57)	*t* = −0.026	4.30 (1.60) 4.38 (1.51)	*t* = −0.334
**Hospital Type**	Non-MOH, Public MOH, Public Teaching Private	41.00 (6.39) 37.00 (7.96) 36.63 (6.08) 39.43 (6.89)	***F*** **= 5.003 ^b^**	30.91 (5.99) 27.83 (5.41) 29.07 (5.14) 29.35 (5.15)	***F*** **= 2.736 ^a^**	4.71 (1.09) 4.52 (0.88) 4.70 (0.95) 4.95 (1.02)	*F* = 1.807	5.46 (1.14) 4.15 (1.34) 5.05 (1.19) 4.64 (1.95)	***F*** **= 7.855 ^c^**	4.30 (1.77) 4.06 (1.30) 4.57 (1.34) 4.43 (1.76)	*F* = 1.213
**Magnet recognition**	Yes No	41.00 (6.39) 37.57 (7.00)	***t*** **= 3.016 ^b^**	30.91 (5.99) 28.78 (5.24)	***t*** **= 2.390 ^a^**	4.71 (1.09) 4.72 (0.96)	*t* = −0.046	5.46 (1.14) 4.66 (1.53)	***t*** **= 3.303 ^b^**	4.30 (1.77) 4.37 (1.47)	*t* = −0.271
**Unit Type**	General ICU ER	38.82 (7.39) 36.65 (7.13) 38.65 (6.20)	*F* = 1.820	28.94 (5.49) 28.67 (5.27) 30.01 (5.51)	*F* = 1.152	4.34 (1.01) 5.15 (0.74) 4.82 (0.82)	***F*** **= 10.604 ^c^**	4.65 (1.63) 5.00 (1.33) 4.94 (1.39)	*F* = 1.284	4.01 (1.68) 4.48 (1.47) 4.79 (1.21)	***F*** **= 5.937 ^b^**
**EBP training**	Yes No	38.32 (6.98) 38.15 (7.09)	*t* = 0.175	29.32 (5.49) 29.00 (5.39)	*t* = 0.410	4.84 (0.98) 4.45 (0.96)	***t*** **= 2.818 ^b^**	4.87 (1.49) 4.71 (1.51)	*t* = 0.774	4.33 (1.54) 4.41 (1.52)	*t* = −0.367
**Research involvement**	Yes No	39.05 (7.21) 37.80 (6.86)	*t* = 1.298	29.85 (5.76) 28.84 (5.24)	*t* = 1.353	4.95 (0.95) 4.58 (0.98)	***t*** **= 2.834 ^b^**	4.81 (1.61) 4.83 (1.42)	*t* = −0.091	4.58 (1.31) 4.23 (1.64)	*t* = 1.677
**Research and EBP Council familiarity**	Yes No	39.27 (6.46) 34.82 (7.74)	***t*** **= 4.131 ^c^**	29.88 (5.30) 26.92 (5.40)	***t*** **= 3.498 ^b^**	4.83 (0.96) 4.33 (0.98)	***t*** **= 3.262 ^b^**	4.99 (1.45) 4.24 (1.53)	***t*** **= 3.214 ^b^**	4.46 (1.52) 4.02 (1.55)	*t* = 1.789

Independent samples *t* test, ANOVA, and Pearson’s correlation were used. ^a^ *p* < 0.05; ^b^ *p* < 0.01; ^c^ *p* < 0.001.

**Table 4:** Hierarchical multiple linear regression of knowledge, attitudes, and implementation regarding EBP among staff nurses (N = 227).

Step	Variable	EBP					
		Knowledge		Attitudes		Implementation	
		β	R^2^ Change	β	R^2^ Change	β	R^2^ Change
1	Demographics *		**0.070 ^a^**		0.017		0.024
	EBP training—Yes	**0.152 ^a^**		0.052		−0.057	
	Research involvement—Yes	**0.160 ^a^**		−0.014		0.125	
2	Level of education—Diploma	**−0.132 ^a^**	**0.277 ^c^**		**0.150 ^c^**		**0.158 ^c^**
	EBP training—Yes	**0.138 ^a^**					
	Organizational factors						
	Magnet recognition—Yes	−0.114		**0.148 ^a^**		−0.077	
	Unit type—ICU	**0.329 ^c^**		**0.156 ^a^**		**0.160 ^a^**	
	Unit type—ER	**0.169 ^b^**		−0.093		**0.228 ^b^**	
	Research and EBP Council familiarity—Yes	0.047		0.104		0.036	
	Nursing leadership support	**0.321 ^c^**		**0.262 ^b^**		**0.218 ^a^**	
	Work environment support	**0.172 ^a^**		0.031		0.144	
3	Magnet recognition—Yes				**0.049 ^c^**		**0.246 ^c^**
	Unit type—ER			**0.179 ^b^**		**0.128 ^a^**	
	Nursing leadership support					0.001	
	Work environment support			0.173		0.063	
	Knowledge			−0.016		**0.418 ^c^**	
	Attitudes			**0.275 ^c^**		**0.318 ^c^**	
**Cumulative R^2^**			**0.347 ^c^**		**0.217 ^c^**		**0.429 ^c^**

^a^ *p* < 0.05; ^b^ *p* < 0.01; ^c^ *p* < 0.001 * Other variables included in model 1: age, gender, nationality, years of experience, and level of education.

## Data Availability

Data available on request due to privacy and ethical restrictions.
